# Band Structure Engineering of Bi_4_O_4_SeCl_2_ for Thermoelectric Applications

**DOI:** 10.1021/acsorginorgau.2c00018

**Published:** 2022-07-14

**Authors:** Jon A. Newnham, Tianqi Zhao, Quinn D. Gibson, Troy D. Manning, Marco Zanella, Elisabetta Mariani, Luke M. Daniels, Jonathan Alaria, John B. Claridge, Furio Corà, Matthew J. Rosseinsky

**Affiliations:** † Department of Chemistry, Materials Innovation Factory, University of Liverpool, 51 Oxford St, Liverpool L7 3NY, United Kingdom; ‡ Department of Chemistry, University College London, 20 Gordon St, Kings Cross, London WC1H 0AJ, United Kingdom; § Department of Earth, Ocean, and Ecological Sciences, University of Liverpool, 4 Brownlow St, Liverpool L69 3GP, United Kingdom; ∥ Department of Physics, University of Liverpool, Oxford St, Liverpool L69 7ZE, United Kingdom

**Keywords:** thermoelectric, electronic structure, doping, screening, multianion, disproportionate, Bi_4_O_4_SeCl_2_

## Abstract

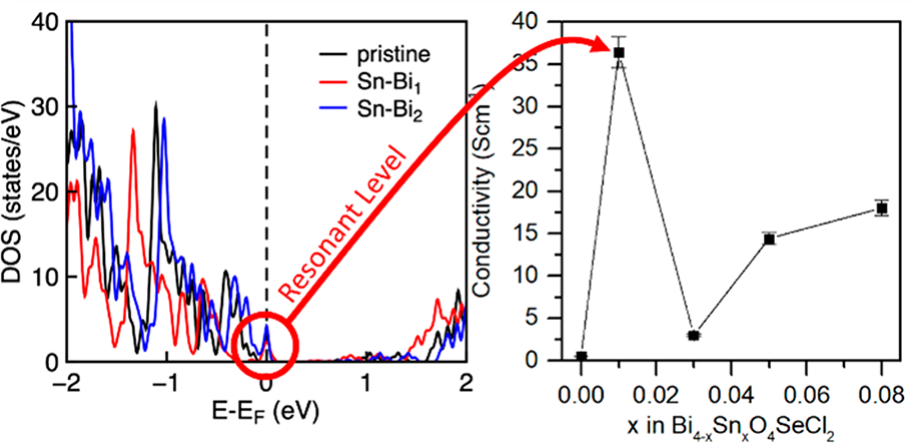

The mixed anion material Bi_4_O_4_SeCl_2_ has an ultralow thermal conductivity of 0.1 W m^–1^ K^–1^ along its stacking axis (*c* axis) at room temperature, which makes it an ideal candidate for electronic band structure optimization via doping to improve its thermoelectric performance. Here, we design and realize an optimal doping strategy for Bi_4_O_4_SeCl_2_ from first principles and predict an enhancement in the density of states at the Fermi level of the material upon Sn and Ge doping. Experimental work realizes the as-predicted behavior in Bi_4–*x*_Sn_*x*_O_4_SeCl_2_ (*x* = 0.01) through the precise control of composition. Careful consideration of multiple accessible dopant sites and charge states allows for the effective computational screening of dopants for thermoelectric properties in Bi_4_O_4_SeCl_2_ and may be a suitable route for assessing other candidate materials.

## Introduction

The global demand for energy has resulted in significant environmental damage, leading to governments setting strict targets for a reduction in fossil-fuel-derived energy consumption and emissions.^1^ As such, numerous technologies have been developed to provide green and renewable electricity or to improve the efficiency of incumbent technologies.^2^ Thermoelectric generators are all solid-state devices that convert a thermal gradient directly into a potential difference and can be utilized to convert the waste heat from devices into useable energy.^3,4^ They are silent, reliable, and scalable, making them ideal for small scale power generation. Despite these benefits, thermoelectric generators are not widely used in industry because of their low conversion efficiencies (typically around 5%).^3^ The thermoelectric figure of merit (*zT*) of a material determines its performance for use in a thermoelectric generator:
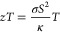


At an absolute temperature (*T*), the *zT* of a material is calculated from its electrical conductivity (σ), thermal conductivity (κ), and Seebeck coefficient (*S*). A material’s *zT* can be improved by increasing the power factor (*PF* = *σS*^2^), or by minimizing κ. Maximising a material’s *zT* requires careful tuning of these properties because they are coupled by the carrier concentration and carrier effective mass.^5^

Doped bismuth chalcogenide materials such as Bi_2_O_2_Se, BiCuSeO, and Bi_2_Ch_3_ (Ch = S, Se, Te) are known to exhibit good thermoelectric figures of merit (*zT* = 0.3–1.3) because of their low thermal conductivities (0.8–1.4 W m^–1^ K^–1^).^6−11^ Bi_4_O_4_SeCl_2_ is a recently reported material with a unique structure; it has a repeating ABCBA van der Waals layered structure consisting of a 2:1 superlattice of BiOCl and Bi_2_O_2_Se.^12^ There is interlayer mixing of the chlorine and selenium atoms, and the material behaves as a semiconductor with an indirect optical band gap of 1.15 eV. Bi_4_O_4_SeCl_2_ exhibits the lowest thermal conductivity of any inorganic crystalline solid with values of 0.4 (1) and 0.10 (2) W m^–1^ K^–1^ in the in-plane (*ab*) and out-of-plane (*c*) directions of a densified and highly textured pellet, respectively, at room temperature.^13^ Because of this outperformance, Bi_4_O_4_SeCl_2_ is an ideal candidate for the measurement and optimization of its thermoelectric performance by doping.

Finding a doping strategy that results in the greatest improvement of properties for a new material can be an arduous task because of the large number of potential dopants and dopant concentrations.^14−20^ As such, numerous approaches for computationally screening dopants have been investigated.^21−23^ Here, we calculate the formation energies of Bi_4_O_4_SeCl_2_ with a range of candidate dopant elements on different crystallographic sites in order to gain an understanding of their stability and likely site occupancy within the parent material. We use this to develop an optimal doping strategy by calculating the effective band structures of Bi_4_O_4_SeCl_2_ substituted with each candidate dopant on the most stable site to observe if any produce modifications to the band structure form that are favorable for thermoelectric applications. We investigate Na, K, Mg, Ca, Sr, and Ba since Group I and II metals have been observed to improve the electrical properties and the degree of phonon scattering in bismuth chalcogenide materials.^18,24−27^ We also calculate the band structure of Sn-doped Bi_4_O_4_SeCl_2_ as Sn doping is known to produce resonant donor levels in bismuth chalcogenides such as Bi_2_Te_3_.^28^

Resonant impurity levels are important phenomena that can be used to increase the magnitude of the Seebeck coefficient and electrical conductivity in thermoelectric materials.^28−30^ For example, the *zT* of SnTe can be more than doubled by resonant level doping with Zn or In.^30−32^ For “normal” dopants, we consider impurity atoms creating donor or acceptor levels below the conduction band minimum (CBM) or above the valence band maximum (VBM), respectively. In contrast, a resonant donor impurity can be considered as creating a bound level with an energy that hybridizes with the states at the edges of the conduction or valence bands to cause a peak in the density of states (DOS) at the Fermi level.^33,34^ This is particularly useful for thermoelectric materials because it leads to an increase in the effective carrier mass and, in turn, the Seebeck coefficient, as the two are related by the Mott equation:
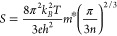
where *S* is the Seebeck coefficient, m* is the carrier effective mass, *n* is the carrier concentration, and *T* is the temperature.^5^ As such, methods to decouple the electronic conductivity and Seebeck coefficient, such as resonant level doping, are invaluable.^35^

The second type of band structure modification we focus on is the formation of in-gap impurity states. Like resonant level doping, the formation of in-gap impurity states also forms a characteristic peak in the DOS at the Fermi level.^36^ However, in this case, the impurity atom forms an isolated state in the band gap of the parent material rather than resonating with the conduction or valence bands. This is advantageous for thermoelectric properties because the isolated state behaves as a source of charge carriers that can be excited and pins the Fermi level in a favorable position to optimize the power factor of thermoelectric materials.

Because of the attractive properties of materials that display these types of band structure modifications, particularly those containing Sn, we also investigate the other group IV elements (Si, Ge, Pb) since these are also known to improve the properties of bismuth chalcogenide materials.^19,28,37^ Finally, iodine doping is investigated to gain an understanding of how anion doping may affect the properties of Bi_4_O_4_SeCl_2_ as, in the case of Pb_1–*x*_In_*x*_Te_1–*y*_I_*y*_ (where In forms an isolated in-gap state), iodine codoping can be used to further optimize the location of the Fermi level.^38^

From the calculations of stability and band structure, we select dopants that are likely to give enhanced thermoelectric properties for experimental studies and focus on those with resonant levels or in-gap impurity states. In doing so, we demonstrate a 25-fold increase in the *zT* of Bi_4_O_4_SeCl_2_ at 420 K and find that computationally screening dopants for the formation of resonant levels or in-gap impurity states is an effective way to optimize the thermoelectric power factor in low thermal conductivity materials such as Bi_4_O_4_SeCl_2_.

## 2 Results and Discussion

### 2.1 Formation Energies and Density of States

**Figure 1:**
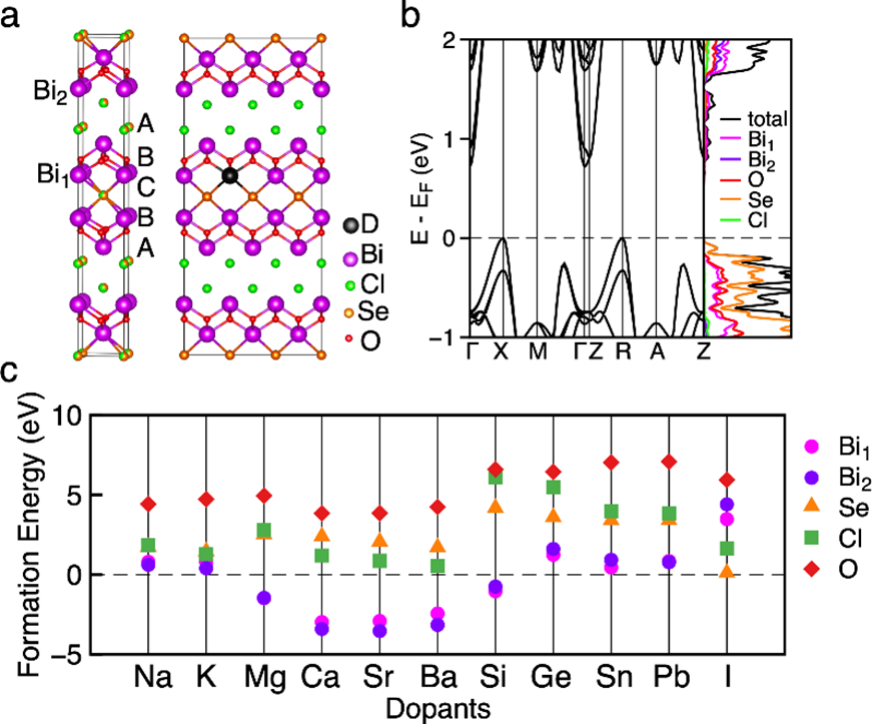
(a) The unit cell of Bi_4_O_4_SeCl_2_ with labels identifying the two bismuth sites and the ABCBA layered structure (left) and the 3 × 3 × 1 supercell of Bi_4_O_4_SeCl_2_ containing a single dopant atom (shown in black) used for DFT calculations (right). (b) The electronic band structure of Bi_4_O_4_SeCl_2_. (c) The predicted formation energies of Bi_4_O_4_SeCl_2_ containing the candidate dopants as obtained from DFT calculations, where the element on the *x* axis is the dopant and the lattice site is defined by the legend.

Figure 1a shows the unit cell of Bi_4_O_4_SeCl_2_ and a 3 × 3 × 1 supercell containing a single dopant atom used for DFT calculations. In the Bi_4_O_4_SeCl_2_ lattice, two fluorite-like [Bi_2_O_2_]^2+^ cation layers are bridged by a Se^2–^ anion layer and are capped with a [Cl_2_]^2–^ layer on each side to form an ABCBA layered structure separated by van der Waals gaps. In reality, the mixing of Cl and Se anions is observed so that it is more appropriate to define anion layers on the basis of their connectivity—as bridging (C), or terminal (A) layers.^12^ The bridging layer is occupied by 52% Se^2–^, while the terminal layer is occupied by 76% Cl^–^. There are two crystallographically distinct Bi sites: Bi (1) is adjacent to the Se-rich bridging atomic layer, while Bi (2) is adjacent to the Cl-rich terminal atomic layer. The electronic band structure and density of states (DOS) of undoped Bi_4_O_4_SeCl_2_ are shown in Figure 1b. For comparison with the doped supercell structures, the unfolded band structure from the undoped 3 × 3 × 1 supercell of Bi_4_O_4_SeCl_2_ is shown in Figure S1 and reproduces the band structure calculated from the single unit cell.

Bi_4_O_4_SeCl_2_ has an indirect band gap where two nearly degenerate valence band maxima (VBM) are located at the X and R points, whereas the conduction band minimum (CBM) is at the Γ point (Figure 1b). The VBM is dominated by Se atomic orbitals, while the CBM is localized in the Bi_2_O_2_ (B) layers with contributions primarily from Bi. The formal valence states of ions in Bi_4_O_4_SeCl_2_ are Bi^3+^, O^2–^, Se^2–^, and Cl^–^, respectively. The formation energies of Bi_4_O_4_SeCl_2_ doped with the candidate dopants are shown in Figure 1c. The elemental state is chosen as the reference for each dopant, and the oxidation state change between the dopant and replaced host-crystal atom is compensated by holes in the valence band or electrons in the conduction band.

For Group I, II, and IV elements, the formation energies for doping onto the Bi (1) and Bi (2) sites are comparable and are the lowest, which indicates that doping will take place to a similar extent on both Bi sites. The lowest formation energies are observed for Group II dopants, thereby indicating that these ions may be the easiest to incorporate into the Bi_4_O_4_SeCl_2_ structure. The formation energy for iodine doping is lowest when it is substituted for selenium, as opposed to a homovalent substitution with chlorine. This may be beneficial for improving the thermoelectric properties in Bi_4_O_4_SeCl_2_ due to the difference in valence states. The oxygen site in the Bi_2_O_2_ layers is inert to substitution with any of the elements considered.

**Figure 2:**
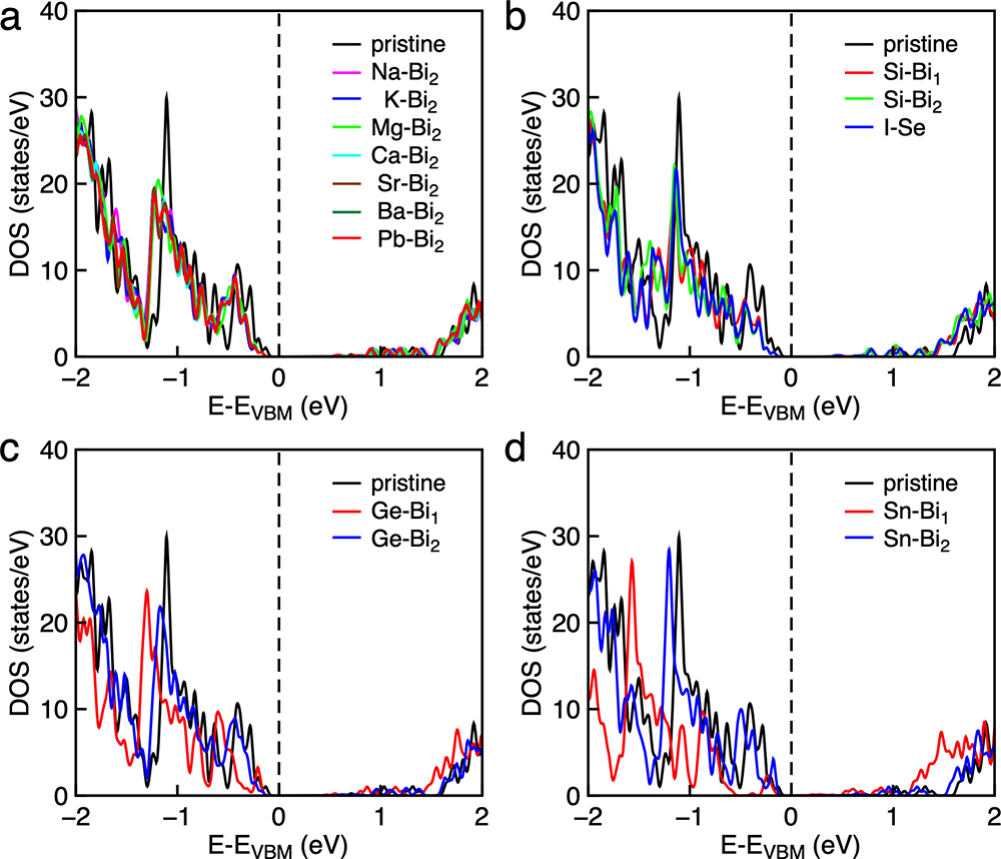
Density of states of (a) Group I (Na, K), Group II (Mg, Ca, Sr, Ba), and Pb-doped Bi_4_O_4_SeCl_2_ at the Bi (2) lattice site; (b) Si-doped Bi_4_O_4_SeCl_2_ at Bi (1) and Bi (2) lattice sites and I-doped Bi_4_O_4_SeCl_2_ at the Se lattice site; (c) Ge-doped Bi_4_O_4_SeCl_2_ at Bi (1) and Bi (2) lattice sites; (d) Sn-doped Bi_4_O_4_SeCl_2_ at Bi (1) and Bi (2) lattice sites.

The band structures and density of states (DOS) were calculated to explore the effects of different dopants on the electronic properties. One dopant atom (*D*) on either bismuth site in the supercell corresponds to compositions of Bi_4–*x*_*D*_*x*_O_4_SeCl_2_ (*x* = 0.056). The DOS of Group I (Na, K), Group II (Mg, Ca, Sr, Ba), and Pb-doped systems were calculated with the dopant element on the more favorable Bi (2) doping site (Figure 2a) according to the predicted formation energies.

The various DOS in Figure 2 are plotted against *E*–*E*_vbm_ to highlight how the DOS is changed by the dopant regardless of the shift in the Fermi energy. Dopants D1 and D2 from Groups I and II, respectively, preferentially replace Bi, induce no significant change in the DOS, and result in p-type behavior (Figure 2a). In Kröger–Vink notation, these dopants are described as D1_Bi_^″^ and D2_Bi_^′^, respectively, whose charge is balanced by the formation of holes in the valence band. Silicon and iodine are found to be n-type dopants described as Si_Bi_^•^ and I_Se_^•^, respectively, and also result in no significant change in the DOS (Figure 2b).

**Figure 3:**
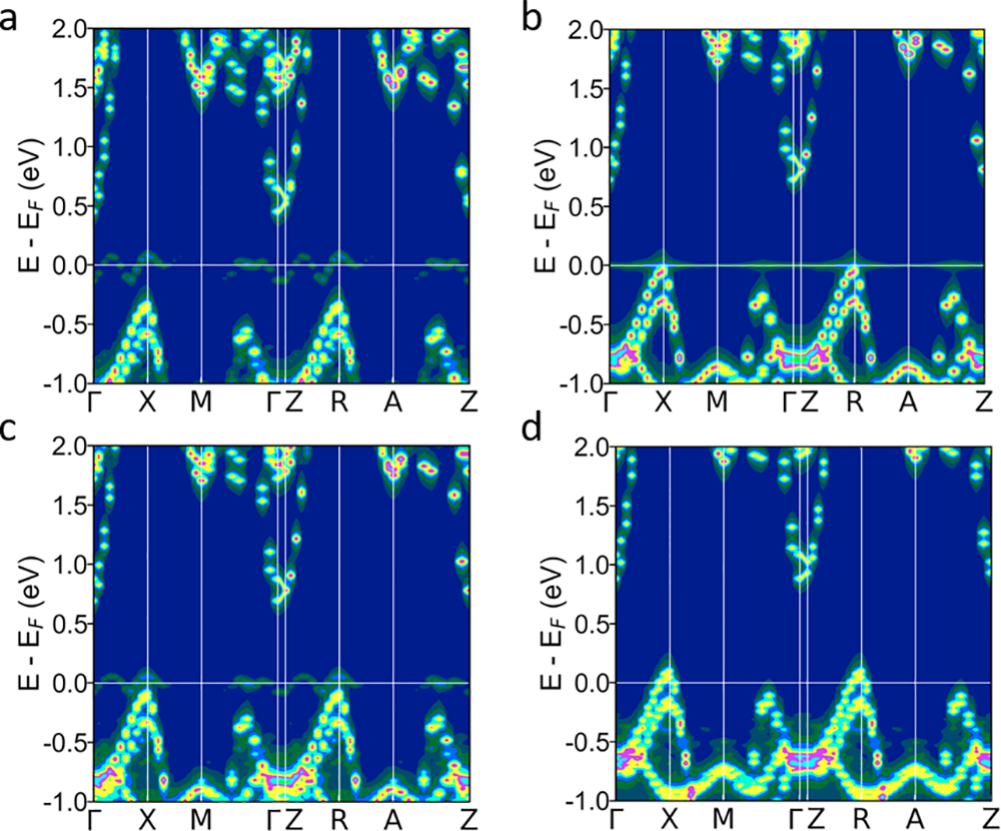
Unfolded effective band structures of doped Bi_4_O_4_SeCl_2_ using 3 × 3 × 1 supercells containing a single (a) Sn_Bi (1)_, (b) Sn_Bi (2)_, (c) Ge_Bi (1)_, or (d) Ge_Bi (2)_ subsitiution.

As also seen in their effective band structures (Figures 3a–d), the heavier Group IV dopants (Ge and Sn) show more complex behavior, with the formation of impurity levels with dopant 4s/5s character in close proximity of the Fermi level. The resulting peak in the DOS is present for Sn substitutions at both the Bi (1) and Bi (2) sites, whereas for Ge, the peak in the DOS only occurs when doping at the Bi (1) site. While at Bi (2), germanium behaves as a normal p-type (Ge_Bi (2)_^′^) 2+ dopant, and the DOS remains largely unchanged. The more electronegative Pb yields p-type doping at both the Bi (1) and Bi (2) sites.

This analysis reveals an amphoteric behavior of Ge and Sn, whose valence s states are close to the Fermi energy of the host material and can yield formal 2+ or 4+ oxidation states depending on the crystalline environment. A sharp peak in the DOS at the band edges has been verified as a feature leading to high thermoelectric performances in materials such as SnTe and PbTe;^30,38^ hence, Ge and Sn were identified as potentially effective dopants for attaining a high thermoelectric power factor in Bi_4_O_4_SeCl_2_. It is difficult to infer properties from these band structures because they are convoluted by the fact that Sn appears to form an in-gap impurity level when doped on the Bi (1) site (Figure 3a), while it hybridizes with the valence band on the Bi (2) site to form a resonant level (Figure 3b).

**Figure 4:**
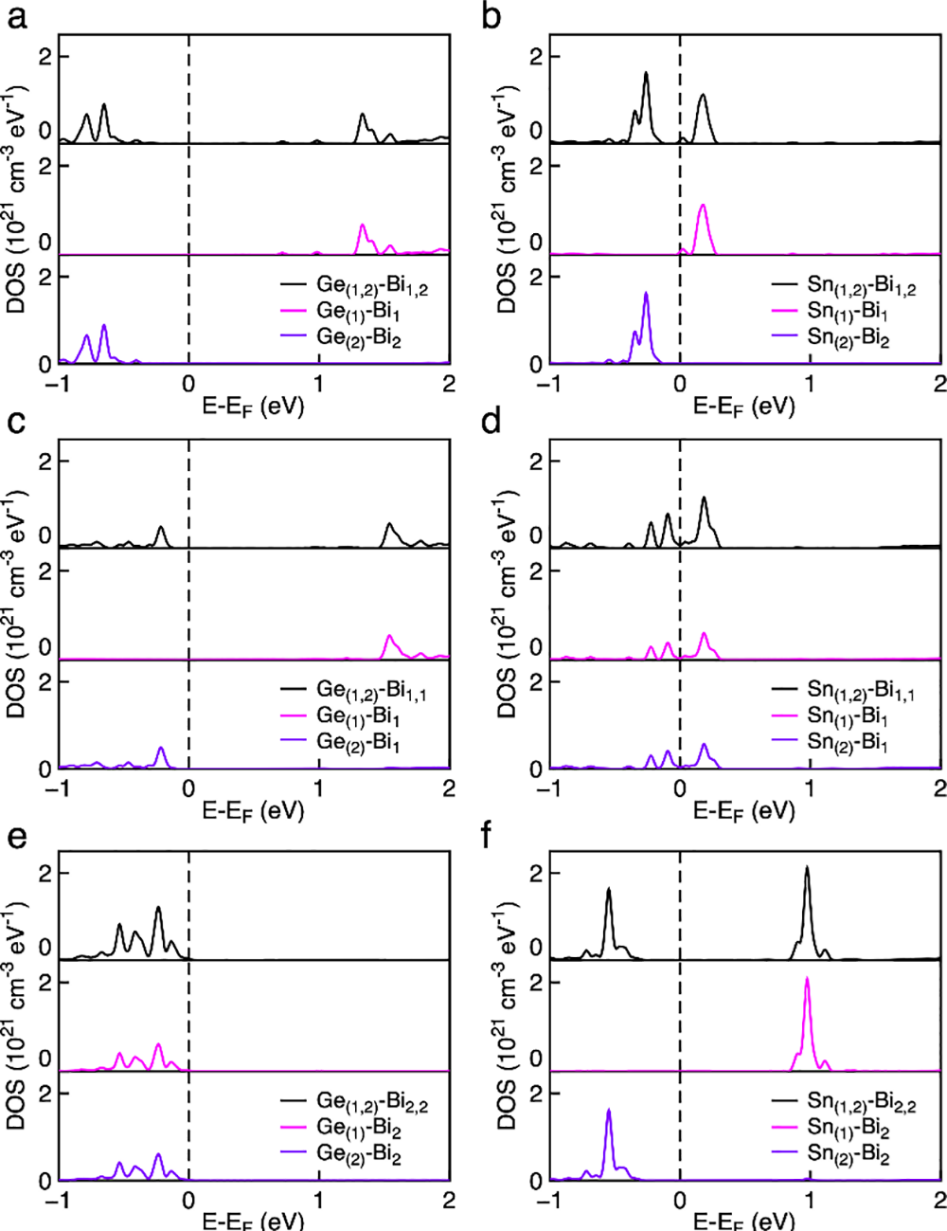
Partial density of states of Bi_4–*x*_*D*_*x*_O_4_SeCl_2_ with the dopant elements on different sites (*D* = Ge, Sn; *x* = 0.112). The doping sites are (a) Ge_Bi (1)_ and Ge_Bi (2)_, simultaneously; (b) Sn_Bi (1)_ and Sn_Bi (2)_, simultaneously; (c) two Ge atoms on only Bi (1) sites in the supercell; (d) two Sn atoms on only Bi (1) sites in the supercell; (e) two Ge atoms on only Bi (2) sites in the supercell; and (f) two Sn atoms on only Bi (2) sites in the supercell. For each panel, the top plot (black line) shows the combined DOS of the two dopant atoms, followed by the separate contribution of each of the two dopant atoms (pink and purple lines).

Since the Fermi level of Ge- and Sn-doped systems is pinned at the band representing the valence 4s/5s states, further calculations have been performed including two dopant atoms per supercell to allow for both oxidation and reduction of the dopants to take place, as required by the electronic structure solution. All combinations of Bi (1) and Bi (2) sites have been examined for the two dopants. The contribution from the dopant orbitals to the density of states near the Fermi level (*E* = 0) is highlighted in Figure 4. Sharp peaks appear on either side of the Fermi level and confirm that Ge 4s and Sn 5s states indeed form localized doping bands in an energy region of interest to enhance the thermoelectric properties.

In both Ge and Sn cases, incorporation at the Bi (2) site next to the Cl layer yields dopant levels at lower energies compared with the Bi (1) site next to Se. This is consistent with the relative ionicity of the two environments and the slightly higher bond valence sum of 3.07 at site Bi (1) compared with 3.01 for Bi (2) (Table S1).^39^ For the Ge 4s states, the lower energy of levels of the Bi (2) site are sufficient to stabilize a 2+ oxidation state for both dopant atoms (Figure 4e); if, however, at least one of the dopants occupies the Bi (1) site, the higher energy of the 4s states causes a disproportionation of Ge into one Ge^4+^ and one Ge^2+^ ion, the former always occupying the Bi (1) site (Figure 4a,c). The disproportionation causes the peak at the Fermi level to disappear and to be replaced by two bands on either side of it. The 5s states of Sn are at a higher energy than the 4s states of Ge, hence, the driving force for reduction to the 2+ oxidation state at site Bi (2) is smaller. The different chemical environments of the Bi (1) and Bi (2) sites are sufficient to induce a disproportionation of the two Sn dopants into Sn^2+^ on Bi (2) and Sn^4+^ on Bi (1) (Figure 4b). However, the respective levels are separated by such a small gap (of the order of 0.1 eV) that the resonant behavior may be restored by thermal occupation of the two levels. The enhanced DOS at the Fermi level is present when both Sn impurities are on the Bi (1) sites (Figure 4d), while disproportionation occurs when both Sn impurities are on the Bi (2) sites (Figure 4f).

This type of behavior is generally atypical in resonant level doping as, for example, changing the doping concentration in In-doped BiCuSeO only affects the weight of the band projection and does not move the relative energies of the In orbitals away from the Fermi level.^17^ However, somewhat similar behavior has been reported in materials such as the resonant-level-doped Hg_1–*x*_Fe_*x*_Se, since Fe has two electron configurations (d^5^ and d^6^) at sufficiently high doping concentrations.^40^ This occurs in Hg_1–*x*_Fe_*x*_Se because there are two competing effects: (1) the hybridization between the impurity and parent states that form the resonant level and (2) the interimpurity Coulombic interactions, which dominate at low doping concentrations and prevent the iron from disproportionating. This is of note because the mobility of the electrons in Hg_1–*x*_Fe_*x*_Se exhibits a unique trend with *x* that can be modeled by considering the interdopant interactions.^38^

This discussion highlights the importance of dopant type, concentration, and location within the parent lattice of Bi_4_O_4_SeCl_2_, as determined by the chemistry and bonding requirements of the dopant element, all of which are factors that can be controlled through the informed selection of dopants and precisely controlled synthesis. The effect of the Se/Cl anion mixing on the disproportionation is unknown; however, it is expected to reduce the chemical difference between Bi (1) and Bi (2) environments and thus reduce the extent of disproportionation and enhance the occurrence of defect states at the Fermi level. It will also increase the number of possible dopant environments (i.e., surrounded by all Se, by all Cl, or by mixed Se/Cl) and lead to an increase in the range of energies of the impurity states. Because of these competing effects on bands above and below the Fermi level, accurate predictions of the doping type (p or n) are difficult to make.

Because the analysis of the computational results indicated that Sn and Ge have low incorporation energy in Bi_4_O_4_SeCl_2_ and yield doped materials with the most favorable features in the density of states to enhance the thermoelectric figures of merit, Ge- and Sn-doped Bi_4_O_4_SeCl_2_ were selected for experimental investigations.

### 2.2 Synthesis and Properties

**Figure 5:**
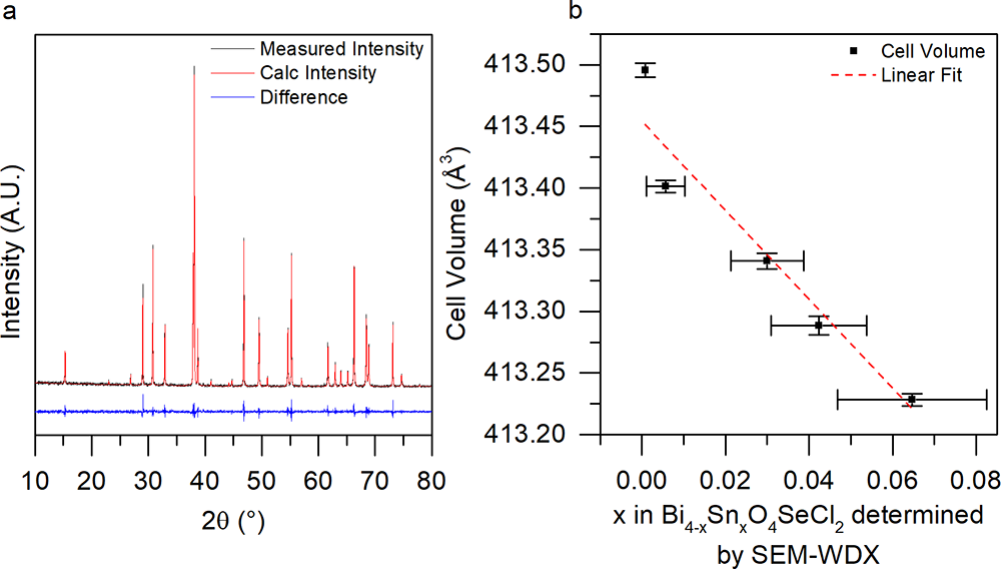
(a) The measured PXRD pattern (black line) and Pawley fit (red line) of single phase Bi_3.92_Sn_0.08_O_4_SeCl_2_. (b) The cell volumes of Bi_4–*x*_Sn_*x*_O_4_SeCl_2_ (0 ≤ *x* ≤ 0.08) samples obtained from Pawley fitting of PXRD data measured with a LaB_6_ internal standard plotted against the Sn content measured by SEM-WDX.

Samples of Bi_4–*x*_Sn_*x*_O_4_SeCl_2_ were synthesized phase pure up to a dopant concentration of 2% (*x* = 0.08), whereas all Bi_4–*x*_Ge_*x*_O_4_SeCl_2_ samples (0.01 ≤ *x* ≤ 0.05) contained a secondary Bi_4_(GeO_4_)_3_ phase in their powder X-ray diffraction (PXRD) patterns (Figure S14). This agrees with Figure 1c, as Ge doping is predicted to require the highest formation energy of any of the assessed dopants. Figure 5a shows a Pawley fit of PXRD data measured from Bi_3.92_Sn_0.08_O_4_SeCl_2_, which represents the material with the highest dopant concentration. The diffraction patterns of Bi_4–*x*_Sn_*x*_O_4_SeCl_2_ (0.01 ≤ *x* ≤ 0.08) show no impurity peaks, and all reflections can be fit with the *I*4/*mmm* space group showing that all doped materials are isostructural with the parent (Figure S13). Above *x* = 0.08, small impurity peaks of BiOCl were observed. The refined unit cell volumes are given in Figure 5b and are observed to follow an almost linear Vegard-type trend as a function of substitution. The decreasing cell volume with increasing Sn content indicates that Sn(IV) is present because it has a smaller ionic radius (0.81 Å) relative to Bi(III) (1.17 Å), whereas the ionic radius of Sn(II) is of similar size (1.22 Å).^41,42^ The Sn content in each sample was measured by scanning electron microscopy wavelength-dispersive spectroscopy (SEM-WDX) and shows the measured composition is within error of the nominal for each dopant concentration.

Phase-pure powders of Bi_4–*x*_Sn_*x*_O_4_SeCl_2_ (0.00 ≤ *x* ≤ 0.08) were processed by spark plasma sintering (SPS) to form dense pellets, which were cut into bars and polished for property measurement. Pole figure data, obtained from electron backscatter diffraction (EBSD) analyses (Figure S16), show the preference for the material to pack with the *c* axis of grains aligning parallel with the pressing direction. This packing preference occurs because plate-like grains (Figure S17) of Bi_4_O_4_SeCl_2_ align perpendicular to the pressing direction, which in turn results from the anisotropy of the structure itself. As a result of the anisotropic properties of Bi_4_O_4_SeCl_2_, changes in the average grain orientation between different samples influence the measured electrical and thermal properties (Figure S18). As such, samples with similar Lotgering orientation factors (*f*) were selected for physical property measurements,^43,44^ where
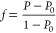
and:
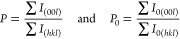
Here, ∑*I*_(*00l*)_ and ∑*I*_0(00*l*)_ are the sums of the integrated PXRD peak intensities of the 00*l* peaks in a measured sample and in a sample with random grain orientation, respectively. ∑*I*_(*hkl*)_ and ∑*I*_0(*hkl*)_ are the sums of the integrated peak intensities of all peaks in a measured sample and in a sample with random grain orientation, respectively. Integrated peak intensities were obtained using a Le Bail intensity extraction. Table 1 lists the orientation factor and relative densities of all samples. Electrical resistivity measurements were made on bars with the pressing direction, and therefore, the stacking direction, perpendicular to the attached current electrodes.

**Table 1:** Calculated Orientation Factors Obtained from Lotgering Analysis of PXRD Data and Densities of Bi_4–*x*_Sn_*x*_O_4_SeCl_2_ Pellets Relative to Their Crystallographic Densities

*x*	orientation factor (*f*)	density (%)
0	0.18	90
0.01	0.20	89
0.03	0.18	89
0.05	0.17	90
0.08	0.14	91

Electrical measurements on Bi_4–*x*_Sn_*x*_O_4_SeCl_2_ (0.00 ≤ *x* ≤ 0.08) were made between 330 and 420 K because measurements at higher temperatures caused an irreversible degradation in electronic conductivity as a result of a surface reaction (Figure S19). The electrical conductivities of samples heated above 420 K can be returned to their previous levels by polishing the faces of the bar postmeasurement.

**Figure 6:**
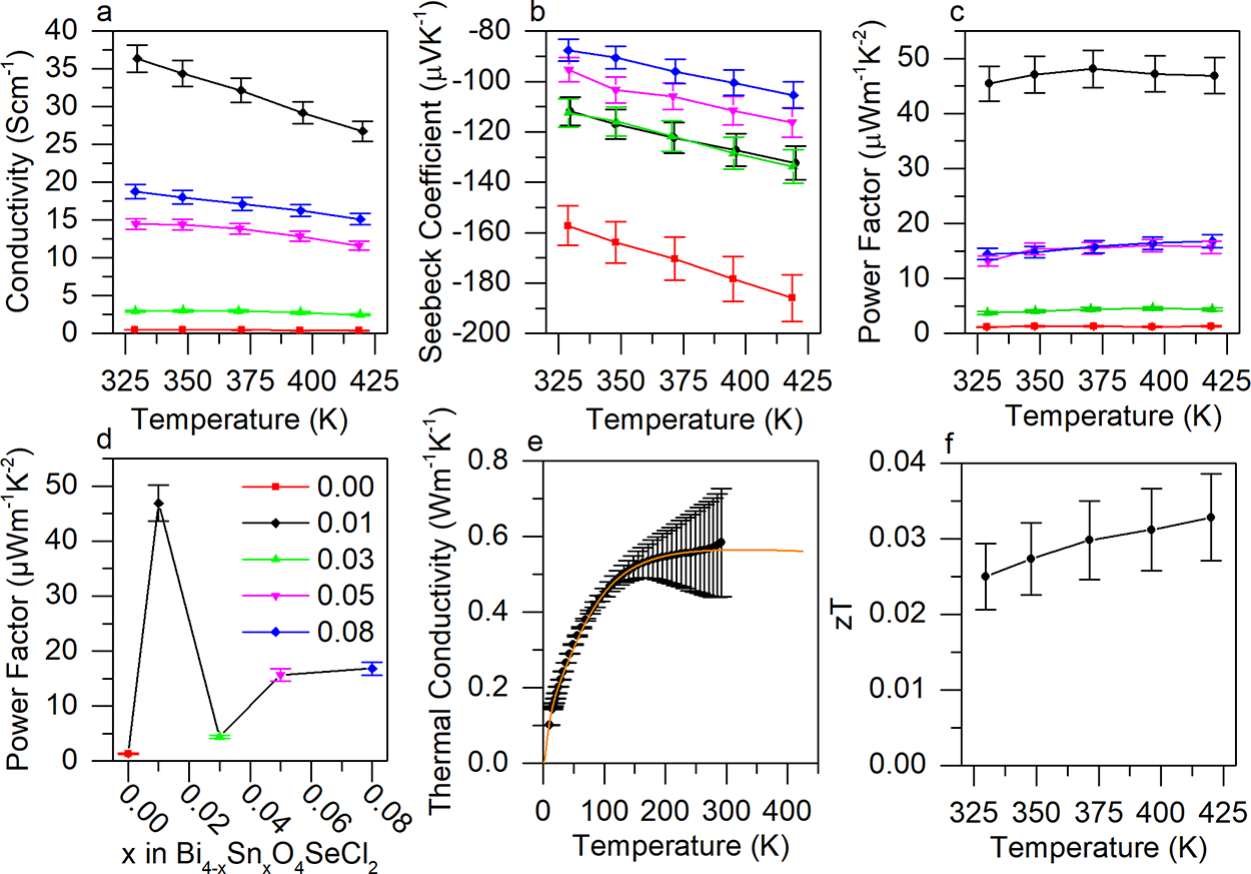
(a) The electrical conductivity, (b) Seebeck coefficient, and (c) power factors (σS^2^) of Bi_4–*x*_Sn_*x*_O_4_SeCl_2_ (0.00 ≤ *x* ≤ 0.08) materials measured in the in-plane (*ab*) direction. (d) The power factors of Bi_4–*x*_Sn_*x*_O_4_SeCl_2_ at 420 K. (e) The thermal conductivity (the orange line plots a model of the thermal conductivity up to 420 K so that the *zT* can be estimated at higher temperatures) and (f) *zT* of the best performing sample (Bi_3.99_Sn_0.01_O_4_SeCl_2_) measured in the in-plane (*ab*) direction.

Because of the anisotropic structure and properties of Bi_4_O_4_SeCl_2_, it is important to ensure all properties were measured in the same plane. Figure 6 panels a and b show the electrical conductivities and Seebeck coefficients of all Bi_4–*x*_Sn_*x*_O_4_SeCl_2_ (0.00 ≤ *x* ≤ 0.08) compositions when measured perpendicularly to the pressing direction (*ab*) up to 420 K, as well as unique trends with doping concentration. The electronic conductivity of the Bi_4–*x*_Sn_*x*_O_4_SeCl_2_ (0.00 ≤ *x* ≤ 0.08) series reaches a maximum at *x* = 0.01, increasing by 2 orders of magnitude from 0.47 (2) S cm^–1^ in Bi_4_O_4_SeCl_2_, to 36 (2) *S* cm^–1^ at *x* = 0.01. However, as the Sn content is increased further to *x* = 0.03, the conductivity decreases to 3.0 (1) S cm^–1^, below that of *x* = 0.01 but above the undoped parent material, and then rises again to 18 (1) S cm^–1^ at *x* = 0.08 (Figure S20a). Above 330 K, the electrical conductivities decrease with increasing temperature, which indicates that the carrier concentration is temperature independent and that the materials are in the degenerate semiconductor regime.^45^ Further, because the Seebeck coefficient has a linear relationship with temperature, the carrier concentration also cannot vary significantly with temperature as the two are related by the Mott formula.^5^

Similarly, the Seebeck coefficient rapidly decreases in magnitude at low doping concentrations, plateaus, and then slowly decreases again as the doping concentration is increased further (Figure S20b). The undoped Bi_4_O_4_SeCl_2_ has the largest magnitude of Seebeck coefficient of −157 (8) μV K^–1^ at 330 K, which decreases as the dopant concentration increases from *x* = 0.03 to 0.08 until reaching a minimum value of −88 (4) μV K^–1^. The *x* = 0.01 sample does not fit the expected trend of the Seebeck coefficient increasing proportionally with *x*, as it is within error of the *x* = 0.03 sample at all temperatures (Figure 6b). Because this trend is somewhat unusual, a repeat data set was collected to verify the results and is plotted in Figure S20.

The *x* = 0.01 dopant concentration leads to an increase in electronic conductivity of 2 orders of magnitude compared with the parent. This could be attributed to either the formation of an in-gap impurity level or a resonant donor band as, in each case, it would be a result of the promotion of electrons from the peak in the DOS near the Fermi level to the conduction band. The carrier concentration of Bi_4_O_4_SeCl_2_ is sufficiently small that including Sn dopants simultaneously adds significant charge carriers and moves the Fermi energy in addition to any hybridization or resonant effects.

Despite the large increase in carrier concentration in *x* = 0.01, the Seebeck coefficient only decreases in magnitude to the same degree as the *x* = 0.03 sample, from −186 (9) to −132 (7) μV K^–1^ at 420 K. The observed trend of the electrical conductivity increasing while the Seebeck coefficient remains unchanged has also been reported in the resonant doped materials Bi_6_Cu_2_Se_4_O_6_ and SnSe.^46,47^ In each case, this has been attributed to the formation of a peak in the DOS at the Fermi Level because the increased effective carrier mass of the electrons mitigates the effect of the increased carrier concentration on the Seebeck coefficient (Figure S21).^5^ This indicates a decoupling of these properties at a dopant concentration of *x* = 0.01.^48^ As such, the Bi_3.99_Sn_0.01_O_4_SeCl_2_ sample exhibits a 40-fold increase in power factor (*σ*S^2^) to 47 (4) μW m^–1^ K^–2^ relative to the parent [1.2 (1) μW m^–1^ K^–2^] (Figure 6c). Figure 6d shows the power factors of each sample at 420 K to further illustrate the trend observed, as well as the decoupling of the Seebeck coefficient and electronic conductivity in Bi_3.99_Sn_0.01_O_4_SeCl_2_.

The unusual trends we observe in electronic conductivity and Seebeck coefficient here are consistent with our interpretation of the band structure calculations and may be attributed to the formation of a resonant level or an in-gap impurity level that is stabilized by interdopant Coulombic interactions at low doping concentrations, as the Sn otherwise disproportionates on both the Bi (1) and Bi (2) sites.^40^ This would result in Sn behaving like a typical (nonresonant level) dopant beyond *x* = 0.01 since the effective mass of the electrons are smaller, and fewer impurity states exist near the Fermi level.^34^ This likely occurs because, at sufficiently high dopant concentrations, the distance between the dopant atoms is too small to be mitigated by any interimpurity Coulombic interactions that would dominate over the formation of disproportionated Sn(II) and Sn(IV), similar to iron in Hg_1–*x*_Fe_*x*_Se.^40^

It is necessary to measure both the electronic conductivity and the carrier concentration of the material at low temperatures in comparison to nonresonant level doped materials to elucidate the exact mechanism behind the Sn dopant behavior.^49^ We have attempted to synthesize Bi_4–*x*_Si_*x*_O_4_SeCl_2_, as Si is also predicted to be n-type, however, synthesis of this target yields samples with SiO_2_ impurities. Plotting the nominal carrier concentration (*n*^2/3^) against the Seebeck coefficient gives a linear trend along all samples except for *x* = 0.01, which indicates that this material does not fit with the assumptions that all doped materials have the same effective carrier mass and that this sample does not have nominal carrier concentration (Figure S21).^5^ We were also unable to measure the ratio of Sn(IV) and Sn(II) and the sites they occupy because of the very low doping concentrations (*x* = 0.01 = 0.25%). However, as in Figure 4b, because the gap between the peaks to either side of the Fermi level is narrow (on the order of 0.1 eV), it would be expected that we would continue to observe degenerate semiconductor behavior if the gap is smaller than the thermal energy present.^45^ The n-type behavior of the Sn-doped samples indicates either a larger concentration of Sn(IV) over Sn(II) in the disoproprtionated case or hybridization with the conduction band in the resonant case. While calculations predict hybridization with the valence band to be more likely, a spread in the energy of the Sn states due to Se/Cl mixing could lead these states to hybrdize with the conduction band, as well. The band gaps of all materials are within error of each other and range from 1.20 (5) in the parent to 1.24 (5) in *x* = 0.01 (Figure S22).

The in-plane (*ab*) thermal conductivity of the best performing sample (*x* = 0.01) was measured from 8 to 300 K (Figure 6e). Bi_3.99_Sn_0.01_O_4_SeCl_2_ has a glasslike temperature dependence that plateaus at room temperature around κ = 0.6 (1) W m^–1^ K^–1^. The room temperature in-plane thermal conductivity of the parent is 0.4 (1) W m^–1^ K^–1^.^13^ We used the model developed by Gibson et al. to extrapolate the thermal conductivity to higher temperatures in order to accurately calculate the *zT* of the material at higher temperatures (Figure 6f), which reaches a maximum of 0.033 (6) at 420 K.^13^ While smaller than state-of-the-art room temperature thermoelectric materials such as Bi_2_GeTe_4_ and CuBiSe_2_ (*zT* = 0.2–0.6), this result marks an improvement over Sn-doped Bi_2_O_2_Se (*zT* < 0.01) and is comparable to Ge-doped Bi_2_O_2_Se (*zT* = 0.09) at similar temperatures, which motivates the measurement of Sn-doped Bi_4_O_4_SeCl_2_ in conditions that prevent the surface reaction observed here for future work.^9,50−52^ However, because of the 2D nature of Bi_4_O_4_SeCl_2_, it may have applications in low-temperature thin film devices without the need for extensive modifications.^53^

## 3 Conclusions

The doping effects of Bi_4_O_4_SeCl_2_ with Na, K, Mg, Ca, Ba, Sr, Si, Ge, Sn, Pb, and I have been studied from the perspective of first principles. Group I and II elements and Pb are found to be possible p-type dopants for increasing carrier concentration in Bi_4_O_4_SeCl_2_. Si and I are found to be possible n-type dopants for increasing carrier concentration without affecting the intrinsic n-type thermoelectric charge transport of Bi_4_O_4_SeCl_2_. Peaks at the Fermi level in the DOS are formed when Ge is introduced at the Bi (1) site and when Sn is introduced both at the Bi (1) and Bi (2) sites. Measurements of electrical conductivity and Seebeck coefficient show trends that are consistent with our predictions at low doping concentrations of Sn and highlight this approach as an effective way to decouple the electrical conductivity and Seebeck coefficient in Bi_4_O_4_SeCl_2_ to enable greater control over the power factor. The largest improvement in properties was achieved in the composition Bi_3.99_Sn_0.01_O_4_SeCl_2_, which exhibits an increase of 2 orders of magnitude [from 0.47 (2) to 36 (2) S cm^–1^] in its electronic conductivity, and a 40-fold increase in power factor [from 1.17 (9) to 47 (4) μW m^–1^ K^–1^] compared with the undoped parent at 330 K. Despite the significant increase in electronic properties, the ultralow thermal conductivity of the material is retained. Overall, computational screening for in-gap impurity states and resonant level dopants may be a general strategy for improved thermoelectric performance. However, to ensure confidence in these predictions, additional careful considerations of dopant location and concentration must be made to account for the effects of disproportionation.

## 4 Materials and Methods

### 4.1 Computational Methods

The formation energies and band structures of Bi_4_O_4_SeCl_2_ with different dopants were calculated using density functional theory as implemented in the Vienna ab initio simulation package (VASP).^54,55^ The optB86b-vdW^56^ functional was used to optimize the crystal structures and account for the van der Waals interactions between layers. The cutoff energy of the plane-wave basis set was set to 550 eV. The *k*-mesh in optimization was set to 9 × 9 × 2, and the energy and force convergence were 1 × 10^–5^ eV and 1 × 10^–2^ eV Å^–1^, respectively. Good agreement was obtained between theoretical (*a* = 3.905 Å, *c* = 26.966 Å) and experimental lattice parameters (*a* = 3.8995 (8) Å, *c* = 26.968 (5) Å).^12^ Chemical doping was simulated by building 3 × 3 × 1 supercells comprising 198 atoms on the basis of the optimized structure. Only the Γ point was used in the supercell calculations to relieve the computational load. One dopant atom of Na, K, Mg, Ca, Ba, Si, Ge, Sn, Pb, or I was introduced in the 3 × 3 × 1 supercell, and all symmetry unique positions were considered. The effective band structures for the doped supercells were calculated by using an online code on GitHub, which implements the effective band unfolding technique proposed by Zunger et al., which unfolds the electron band structure in doped supercells to recover an effective band structure on the original unit cell.^57^ Band unfolding has been shown to be a powerful technique for studying doping effects in semiconductors.^58,59^ The doping effects of the Group III, IV, and V elements in BiCuSeO have been analyzed by the same method.^17,19^ The formation energies (*E*_f_) were calculated with the elemental forms of both dopant and lattice atoms replaced, using the stable allotrope at room temperature, according to the following equation:
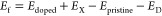
where *E*_doped_ and *E*_pristine_ are the total energies of the doped and pristine supercell, respectively, and *E*_D_ and *E*_X_ are the energies per atom of the dopant and lattice elements in their stable room temperature phase, respectively.

### 4.2 Synthesis and Processing

Bi granules (99.997%), Bi_2_O_3_ (99.9995%), BiOCl (99.999%), GeO_2_ (99.9999%), and SnO_2_ (99.9%) were purchased from Alfa Aesar. Se (≥99.5%) was purchased from Sigma-Aldrich. Bi granules were hand ground into a powder using a pestle and mortar before use.

Bulk samples of Bi_4–*x*_Sn_*x*_O_4_SeCl_2_ and Bi_4–*x*_Ge_*x*_O_4_SeCl_2_ (0.00 ≤ *x* ≤ 0.08) were synthesized by hand grinding powders of Bi, Bi_2_O_3_, BiOCl, and Se with SnO_2_ or GeO_2_ in stoichiometric amounts in an agate pestle and mortar for 10 min. The resulting mixture was sealed in a 6 mm radius quartz ampule that was evacuated to 10^–4^ mbar and fired at 800 °C for 12 h using heating and cooling rates of 5 °C min^–1^. An *x* value of 0.08 corresponds to a maximum doping density of 2%.

Dense pellets (≈90% theoretical density) were obtained by spark plasma sintering (SPS) of the powders at 800 MPa and 400 °C for 5 min in a 10^–3^ mbar vacuum using a commercial Thermal Technology LLC DCS10 furnace. Powder samples (∼1.5 g) were pressed in a 10 mm diameter, graphite-foil-lined, tungsten carbide die set (with 6% Co binder). Heating and pressure ramp rates were set to 50 °C min^–1^ and 100 MPa min^–1^, respectively. The temperature was monitored through a borehole in the side of the die set using a pyrometer. After pressing, the pellets were lightly polished with SiC paper to remove the graphite foil on the pellet surface and were cut into bars with dimensions of 2 × 2 × 7 mm for the Seebeck coefficient, electronic resistivity, and thermal conductivity measurements using a low-speed, diamond-blade saw. The off cuts from the pellets were used for powder diffraction, compositional analysis, and EBSD analyses, from which the pole figures were generated.

### 4.3 Property Measurements

The sample purity was assessed by powder X-ray diffraction (PXRD) using a Panalytical X’Pert PRO diffractometer (Co Kα_1_, λ = 1.788965 Å) with a PIXcel 2D detector in Bragg–Brentano geometry. Pawley fits were performed using TOPAS Academic on PXRD data of samples containing a LaB_6_ (Sigma-Aldrich, 99.5%) internal standard.^60^ The lattice parameters of Bi_4–*x*_Sn_*x*_O_4_SeCl_2_ were refined, the background was modeled using a Chebyshev polynomial function with 12 parameters, and the peak shapes were modeled using a pseudo-Voigt function.

Simultaneous electronic resistivity (ρ) and Seebeck coefficient (*S*) measurements were made on an Ulvac-Riko ZEM-3 instrument from rectangular prism bars. The bars were mounted in a 4-point geometry with the outer current electrodes in contact with each end, and the inner thermocouple and voltage probes were in contact with one of the longer (7 mm) sides of the bar. Au foil was placed between the sample and outer electrodes to prevent any reaction between the two at elevated temperatures. The sample chamber was evacuated and purged three times with helium and then dosed with a 0.01 MPa atmosphere of helium before commencing the measurement. The data were recorded at 25 K intervals from 323 to 423 K, with the application of 10, 20, and 30 K temperature gradients to the bar at each temperature. Errors on both ρ and *S* were assumed to be 5% on advice of the manufacturer.

The thermal conductivity of the bar with the best-performing dopant concentration (Bi_3.99_Sn_0.01_O_4_SeCl_2_) was measured using a four-contact method on a Quantum Design physical properties measurement system (PPMS). The sample was mounted so that the thermal conductivity was measured in the same (*ab*) plane as the electrical conductivity. The thermal conductivity of Bi_3.99_Sn_0.01_O_4_SeCl_2_ was measured from 300 to 8 K with 2 K increments.

The composition of samples was determined by wavelength-dispersive X-ray spectroscopy (WDX) using a Tescan S8000 scanning electron microscope (SEM) equipped with a WDX detector from Oxford Instruments. The detector was calibrated with the appropriate standards for each chemical element. Data acquisition and analysis were performed using INCA software.

The crystallographic orientation of the hot-pressed pellets was obtained using electron backscatter diffraction (EBSD) in a Zeiss GeminiSEM 450 at Liverpool’s Scanning Electron Microscopy Shared Research Facility (SEM SRF). The samples were polished to a high-quality surface finish using chemo-mechanical methods. Data acquisition and analysis were performed using AZtec software. The results are plotted as pole figures in the Supporting Information. For a description of the applications of EBSD to platy crystals, see Dempsey et al.^61^

## Supporting Information Available

Effective band structures of the doped Bi_4_O_4_SeCl_2_, PXRD patterns of all compounds, SEM images, pole figures, and the electrical properties of doped Bi_4_O_4_SeCl_2_ measured in differing conditions (PDF)

## Notes

The underlying data generated in this study can be found at 10.17638/datacat.liverpool.ac.uk/1669.
