# Supplementary material for: Band Structure Engineering of Bi4O4SeCl2 for Thermoelectric Applications
Source: ACS Org Inorg Au. 2022 Jul 14;2(5):405–14. doi: 10.1021/acsorginorgau.2c00018 (PMC9542720; doi:10.1021/acsorginorgau.2c00018)
Supplement: Supplementary file 1 — gg2c00018_si_001.pdf [file gg2c00018_si_001.pdf]

Supporting Information

Band Structure Engineering of $Bi_4O_4SeCl_2$ for Thermoelectric Applications

Jon A. Newnham[a], Tianqi Zhao[b], Quinn D. Gibson[a], Troy D. Manning[a], Marco Zanella[a], Elisabetta Mariani[c], Luke M. Daniels[a], Jonathan Alaria[d], John B. Claridge[a], Furio Corà[b], and Matthew. J. Rosseinsky[a]*

a) Department of Chemistry, Materials Innovation Factory, University of Liverpool, 51 Oxford St, Liverpool L7 3NY, United Kingdom

b) Department of Chemistry, University College London, 20 Gordon St, Kings Cross, London WC1H 0AJ, United Kingdom

c) Department of Earth, Ocean, and Ecological Sciences, University of Liverpool, 4 Brownlow St, Liverpool, L69 3GP United Kingdom

d) Department of Physics, University of Liverpool, Oxford St, Liverpool L69 7ZE, United Kingdom

* corresponding author: rossein@liverpool.ac.uk

Table of Contents

Section	Page
1. **Unfolded Effective Band Structures of doped $Bi_4O_4SeCl_2$**	2
2. **Synthesis and Processing**	7
3. **Property Measurements**	12
4. **References**	16

1. Unfolded Effective Band Structures of doped $Bi_4O_4SeCl_2$ calculated using 3 × 3 × 1 supercells.

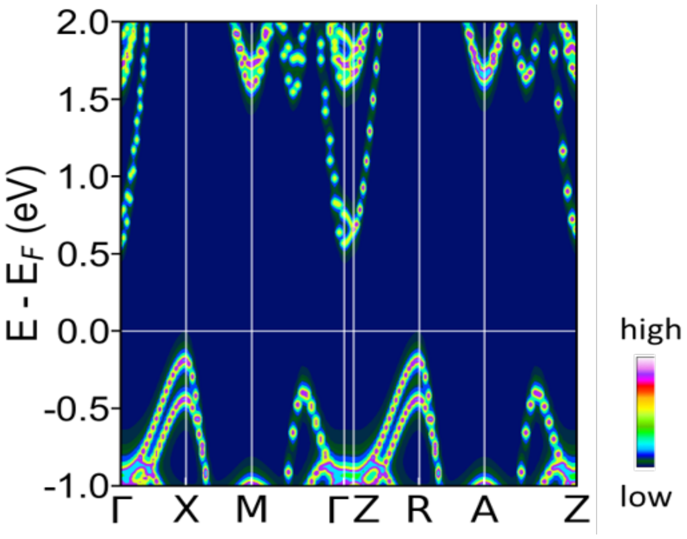

Figure S1. Unfolded effective band structure of pristine $Bi_4O_4SeCl_2$ calculated by using ideal 3 × 3 × 1 supercell.

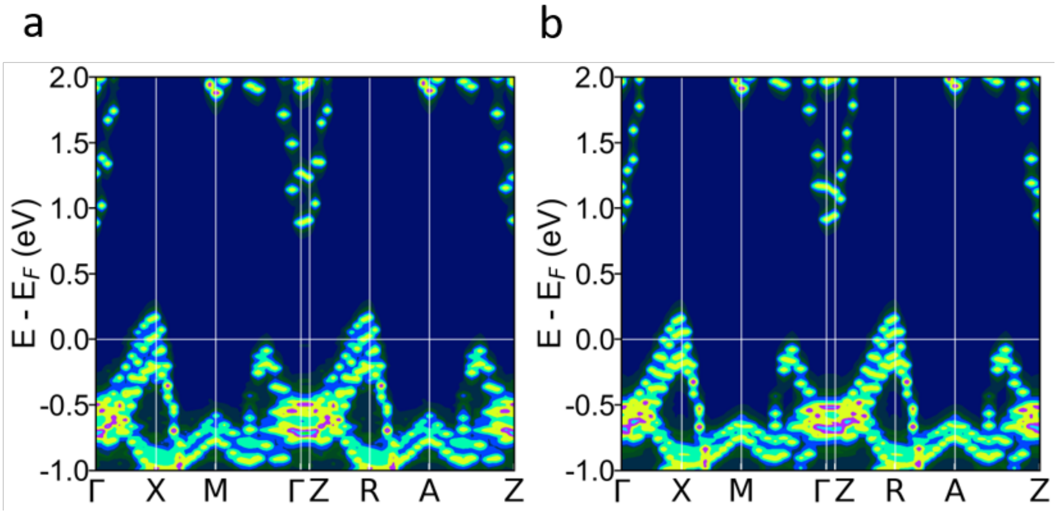

Figure S2. The effective band structure of $B_{3.944}Na_{0.056}O_4SeCl_2$ with a) $Na'_{Bi(1)}$ and b) $Na'_{Bi(2)}$ substitutions only.

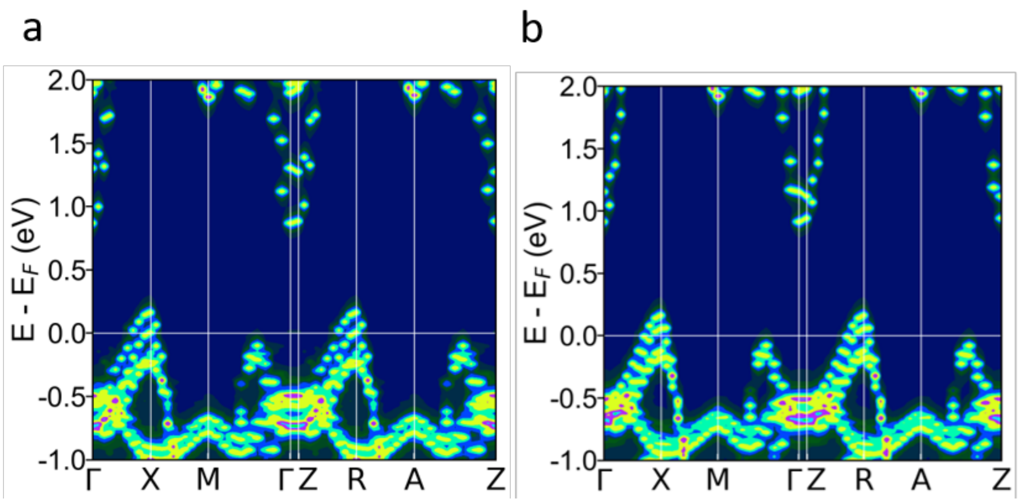

Figure S3. The effective band structure of $B_{3.944}K_{0.056}O_4SeCl_2$ with a) $K^{'}_{Bi(1)}$ and b) $K^{'}_{Bi(2)}$ substitutions only.

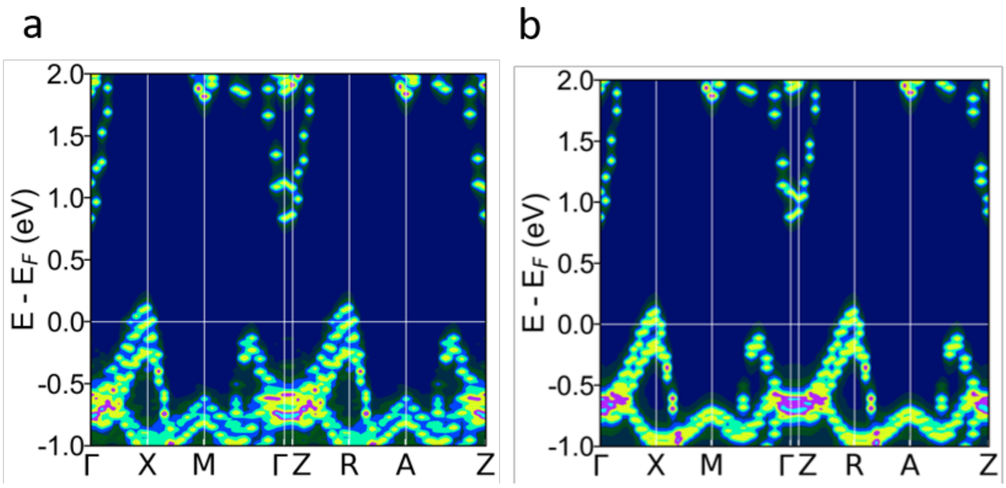

Figure S4. The effective band structure of $B_{3.944}Mg_{0.056}O_4SeCl_2$ with a) $Mg^{'}_{Bi(1)}$ and b) $Mg^{'}_{Bi(2)}$ substitutions only.

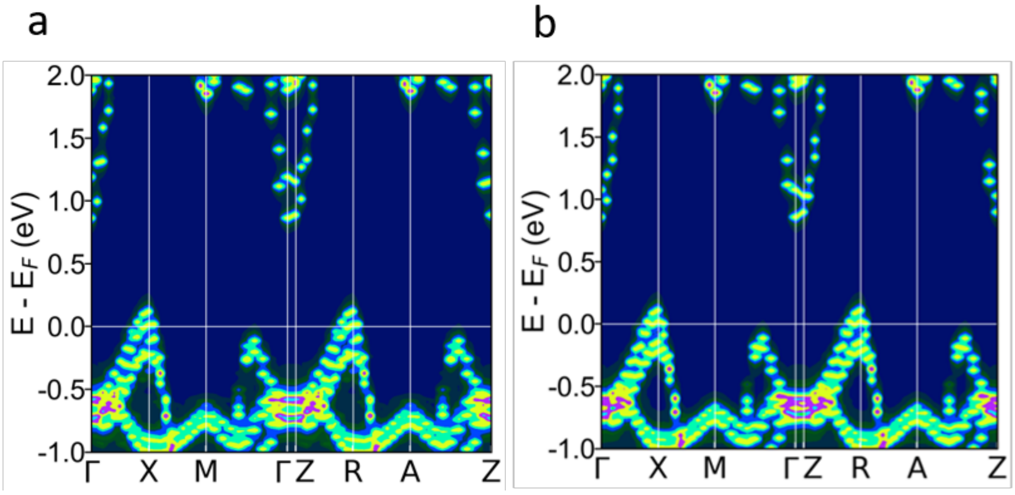

Figure S5. The effective band structure of $B_{3.944}Ca_{0.056}O_4SeCl_2$ with a) $Ca^{'}_{Bi(1)}$ and b) $Ca^{'}_{Bi(2)}$ substitutions only.

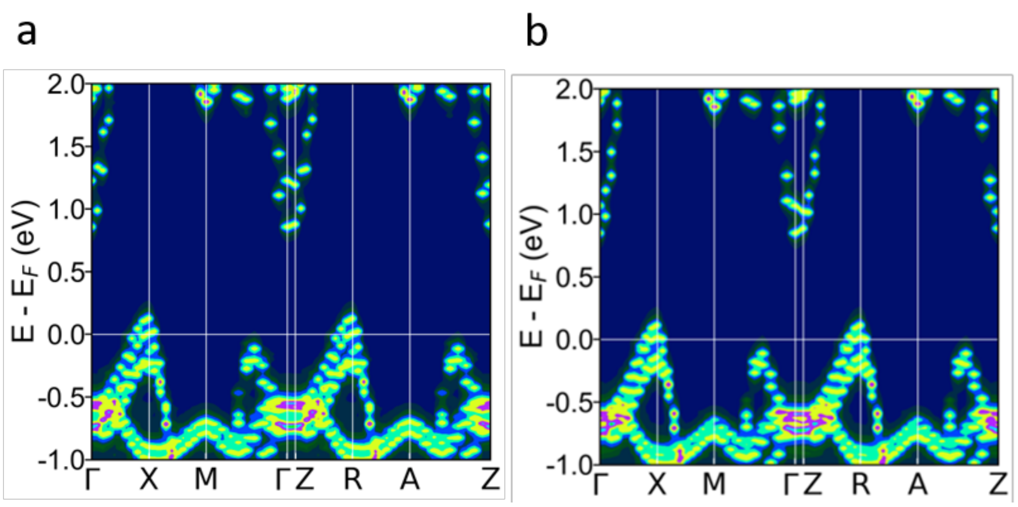

Figure S6. The effective band structure of $B_{3.944}Sr_{0.056}O_4SeCl_2$ with a) $Sr_{Bi(1)}^{'}$ and b) $Sr_{Bi(2)}^{'}$ substitutions only.

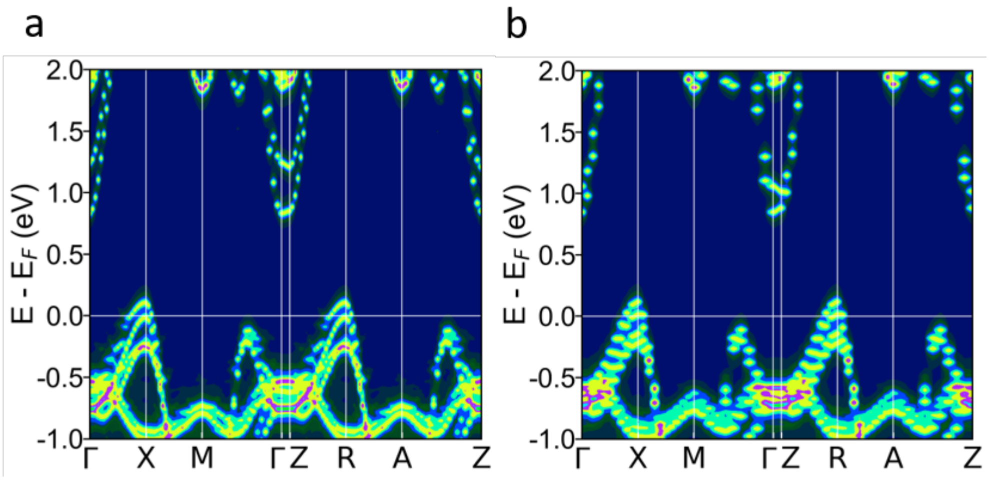

Figure S7. The effective band structure of $B_{3.944}Ba_{0.056}O_4SeCl_2$ with a) $Ba_{Bi(1)}^{'}$ and b) $Ba_{Bi(2)}^{'}$ substitutions only.

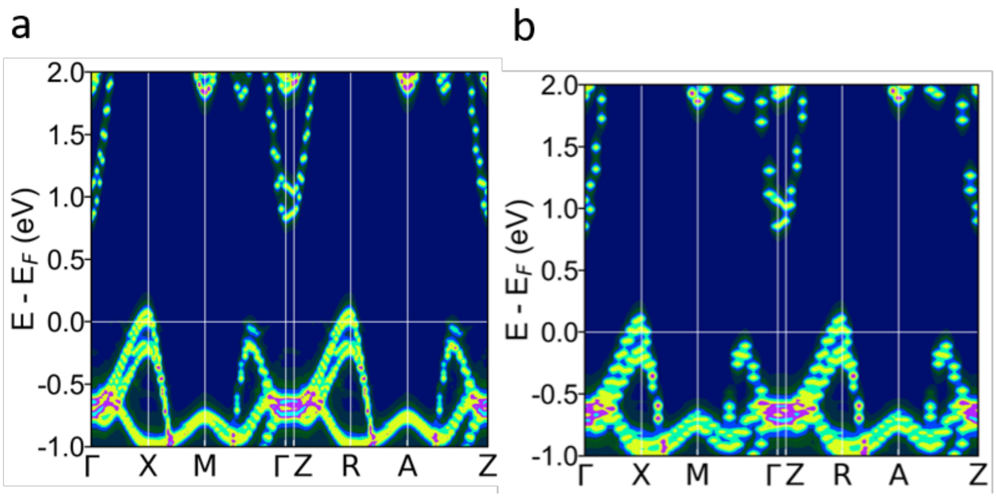

Figure S8. The effective band structure of $B_{3.944}Pb_{0.056}O_4SeCl_2$ with a) $Pb_{Bi(1)}^{'}$ and b) $Pb_{Bi(2)}^{'}$ substitutions only.

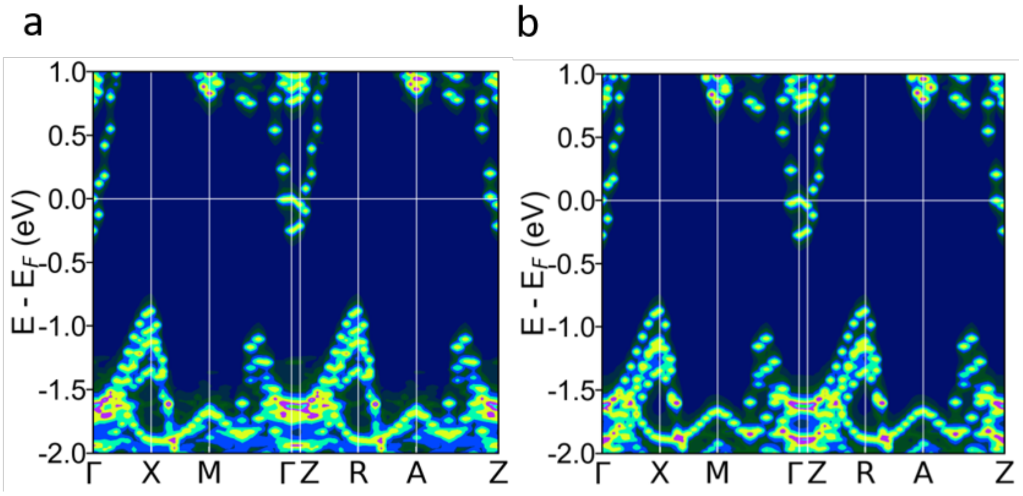

Figure S9. The effective band structure of $B_{3.944}Si_{0.056}O_4SeCl_2$ with (a) $Si^{\bullet}_{Bi(1)}$, and (b) $Si^{\bullet}_{Bi(2)}$ substitutions only.

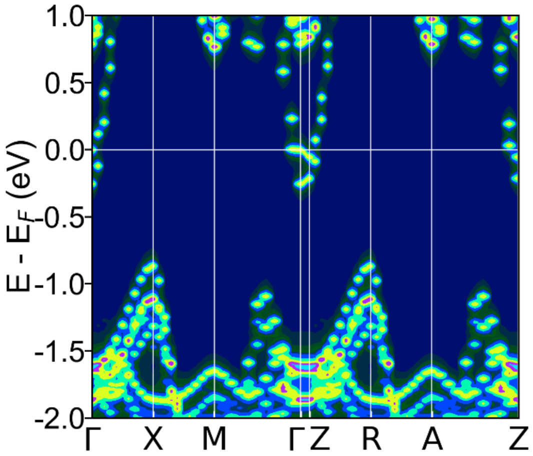

Figure S10. The effective band structure of $B_4O_4Se_{0.944}I_{0.056}Cl_2$.

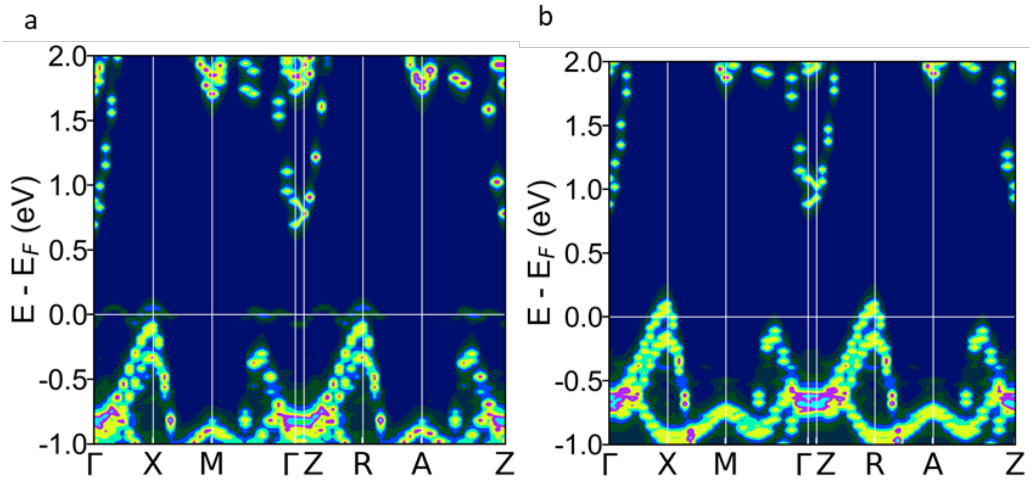

Figure S11. The effective band structure of $B_{3.944}Ge_{0.056}O_4SeCl_2$ with (a) $Ge_{Bi(1)}$, and (b) $Ge_{Bi(2)}$ substitutions only.

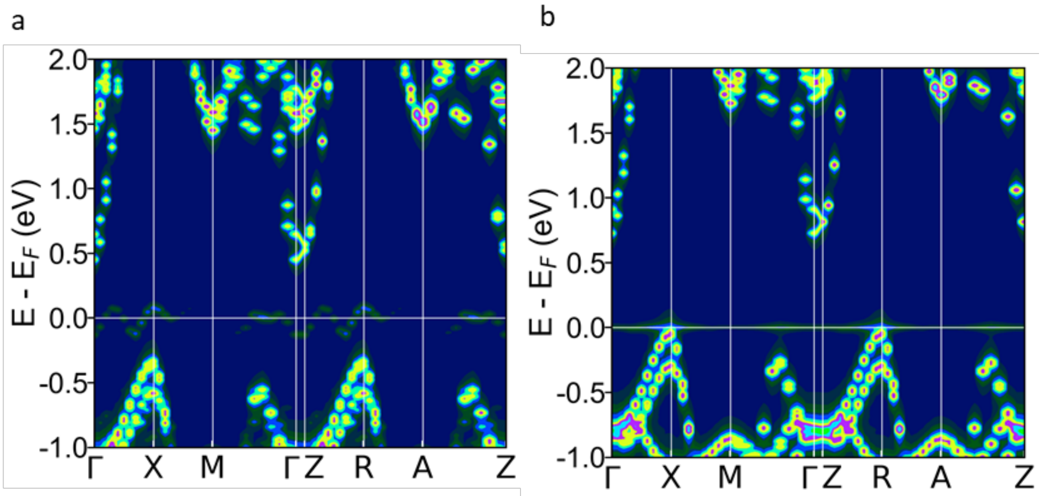

Figure S12. The effective band structure of $B_{3.944}Sn_{0.056}O_4SeCl_2$ with (a) $Sn_{Bi(1)}$, and (b) $Sn_{Bi(2)}$ substitutions only.

2. Synthesis and Processing

Table S1. The lengths and valance parameters of the bonds in $Bi_4O_4SeCl_2$.

	Bi(1)-O	Bi(1)-Se	Bi(1)-Cl	Bi(2)-O	Bi(2)-Se	Bi(2)-Cl
R_0	2.09	2.72	2.48	2.09	2.72	2.48
d_{ij}	2.272	3.3085	3.3085	2.359	3.041	3.041

The bond valance sums for the two bismuth sites were calculated using the equation:

$$V_i = \sum_j \exp\left(\frac{R_0 - d_{ij}}{B}\right) \quad \text{Eq. S1}$$

Where V_i is the bond valance sum of atom *i*, R_0 is the bond-valence parameter between atoms *i* and *j*, d_{ij} is the observed bond length between atoms *i* and *j*, and B is a constant of 0.37. The bond-valence parameters and experimental bond lengths were obtained from Ref. 1 and 2, respectively.

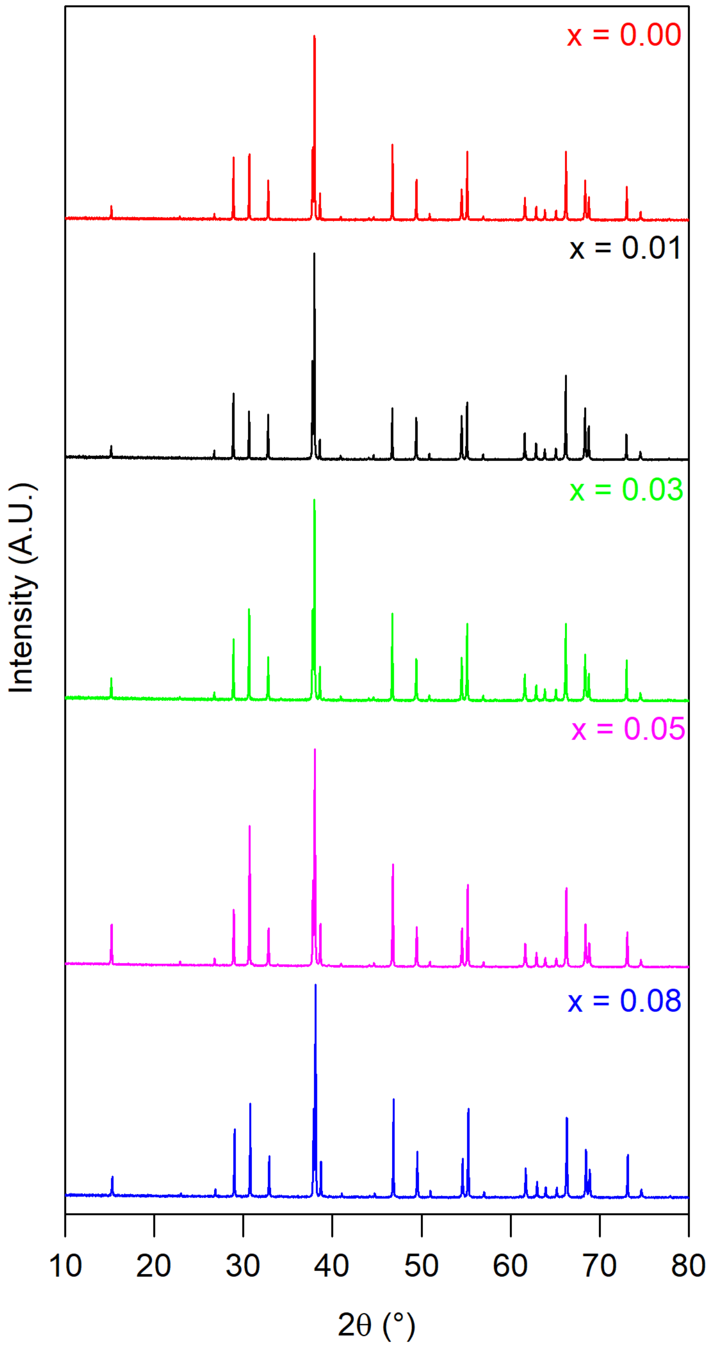

Figure S13. PXRD patterns of $Bi_{4-x}Sn_xO_4SeCl_2$ ($0 \leq x \leq 0.08$).

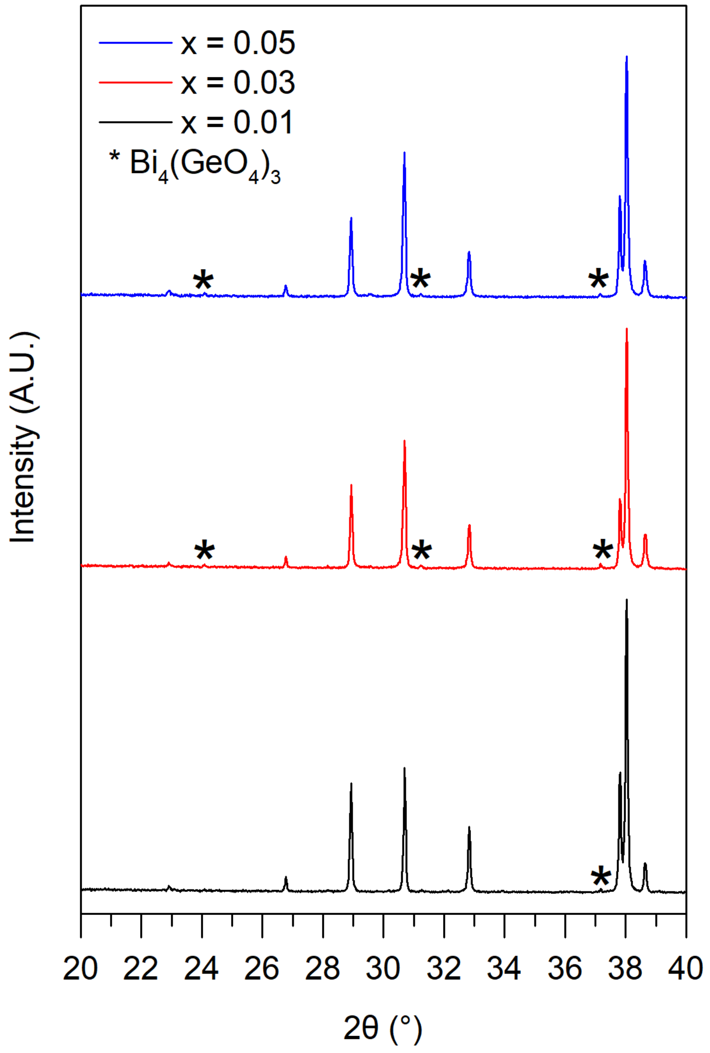

Figure S14. PXRD patterns from the attempted synthesis of $Bi_{4-x}Ge_xO_4SeCl_2$ up to x = 0.05. Impurity peaks of $Bi_4(GeO_4)_3$ are labelled (*).

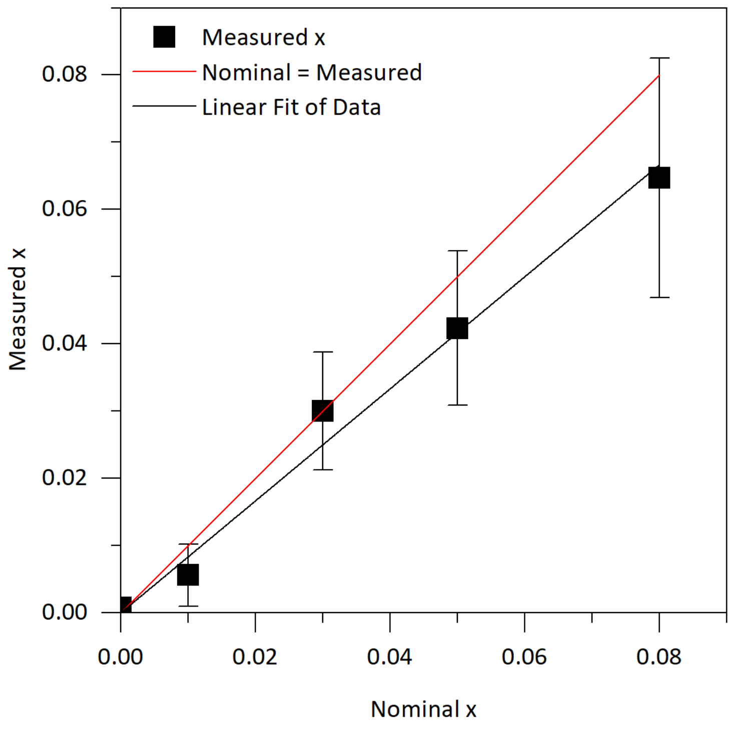

Figure S15. Nominal vs measured compositions of $Bi_{4-x}Sn_xO_4SeCl_2$ when measured by SEM-WDX.

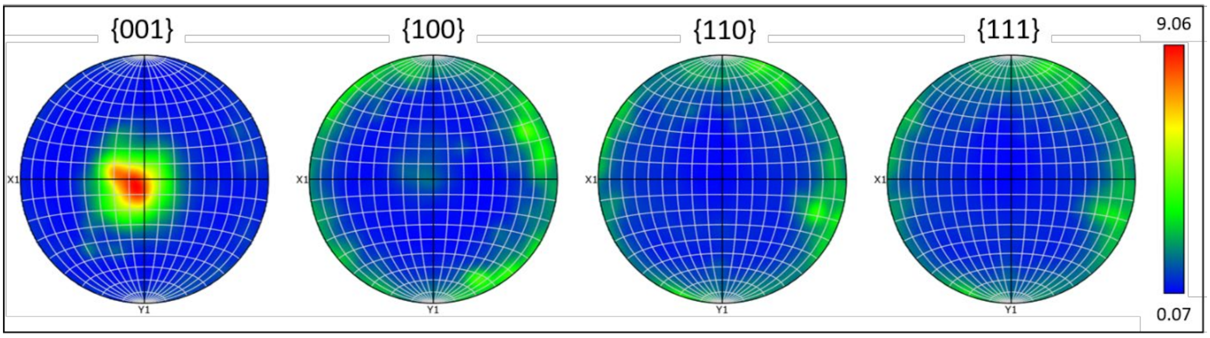

Figure S16. Contoured pole figure data of $Bi_{3.99}Sn_{0.01}O_4SeCl_2$, obtained from electron backscatter diffraction (EBSD) analyses, showing the material's preference to pack with the c-axis of grains aligning parallel with the pressing direction. This is evidenced by the strong maximum (red) in the {001} pole figure. Pole figures are equal area, upper hemisphere projections (in XY) plotted using 10° half width. The colour-coded scale bar on the right (blue to red) represents multiples of uniform density (MUD), used for contouring.

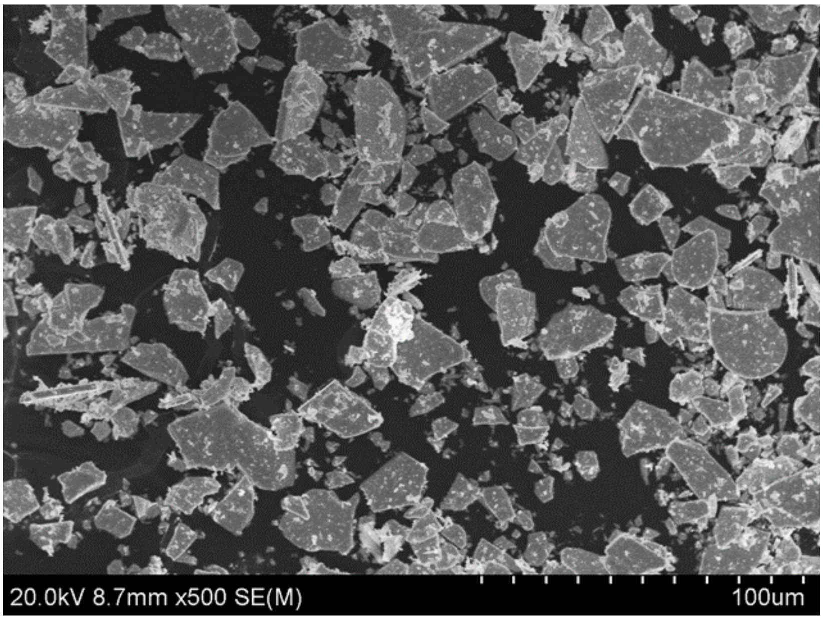

Figure S17: SEM image of the plate like grains in $Bi_{3.99}Sn_{0.01}O_4SeCl_2$

3. Property Measurements

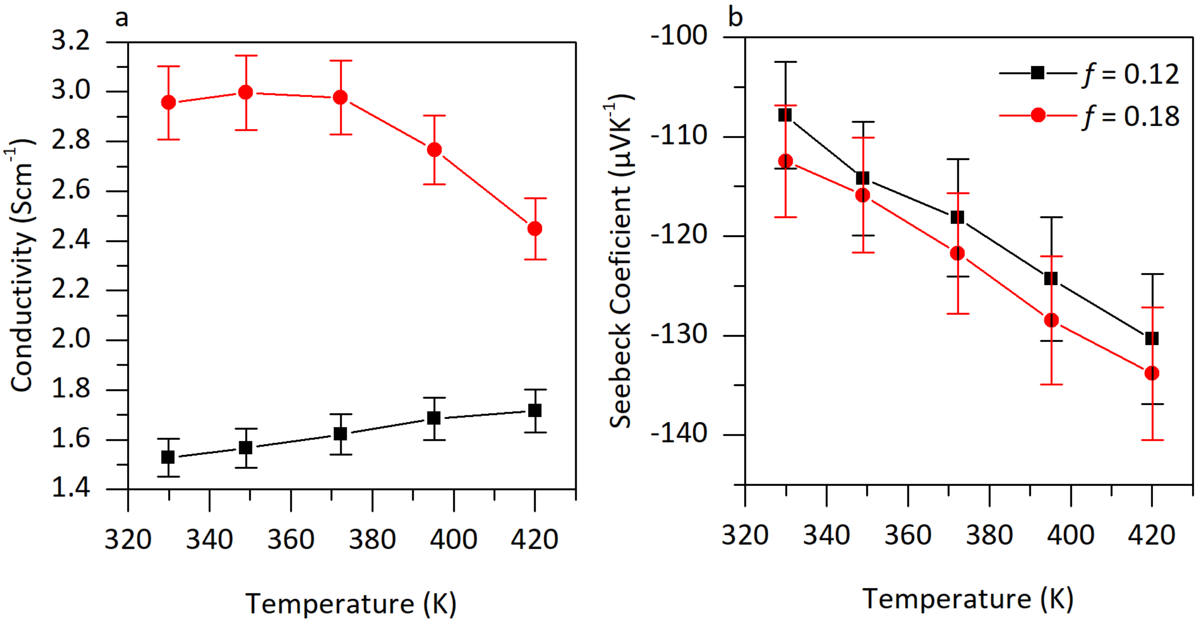

Figure S18. The (a) electrical conductivity and (b) Seebeck coefficients of two pellets pressed of the same $Bi_{3.97}Sn_{0.03}O_4SeCl_2$ sample with different Lotgering orientation factors (*f*).

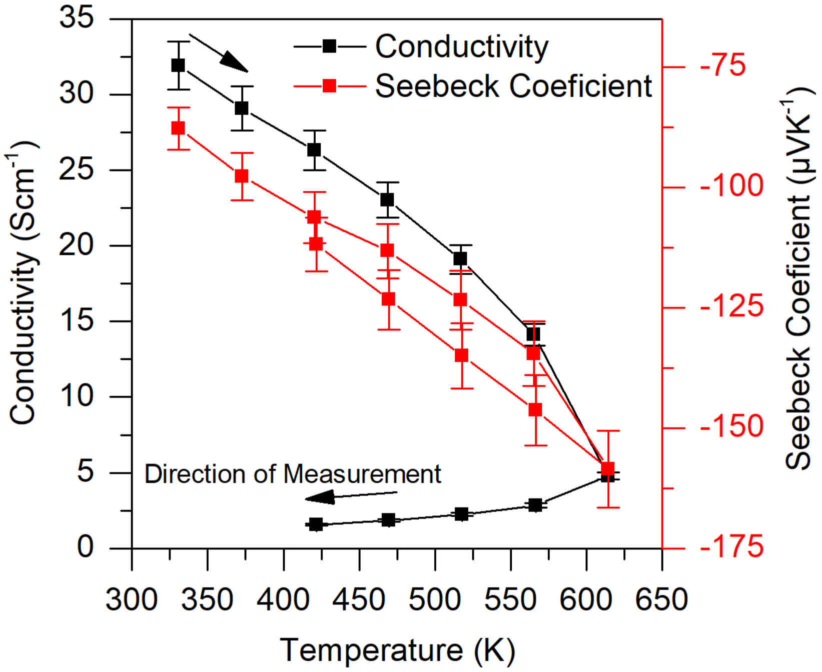

Figure S19. The electrical conductivity and Seebeck coefficients of $Bi_{3.99}Sn_{0.01}O_4SeCl_2$ when heated above 420 K. Samples were measured in an evacuated chamber that was dosed with a 0.01MPa atmosphere of helium. Degradation in electronic conductivity, and not the Seebeck coefficient, indicates that there is not a change to the material bulk. The electronic conductivity can be returned to the pre-heating value by polishing the surface of the bar.

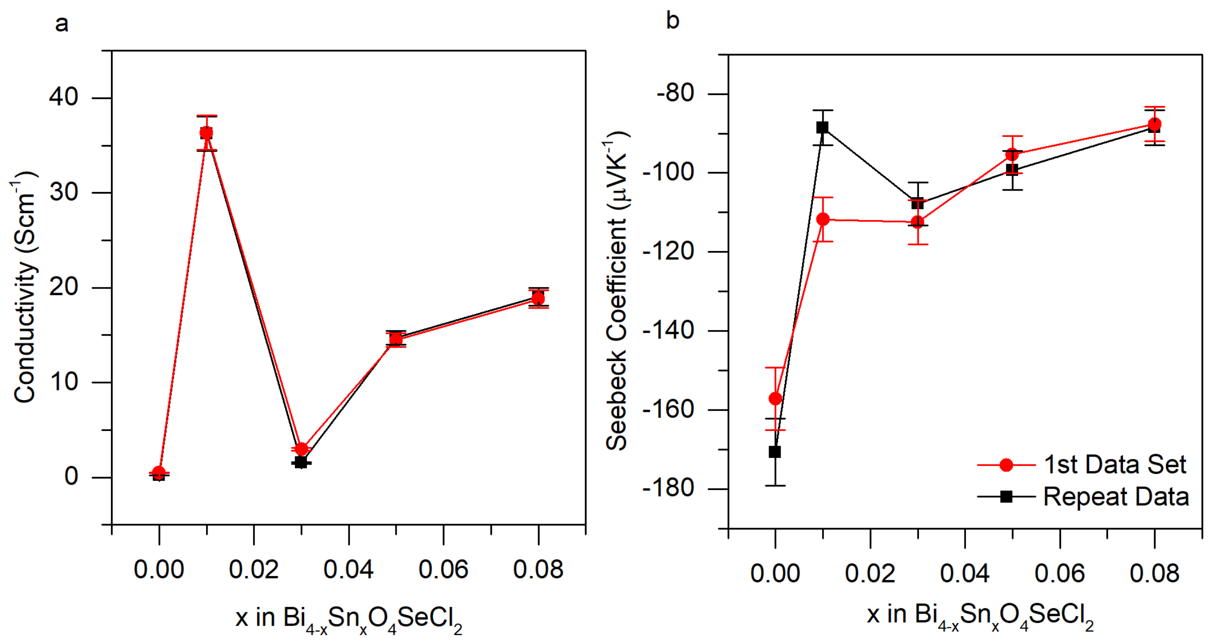

Figure S20. The a) electrical conductivity and b) Seebeck coefficient of two different $Bi_{4-x}Sn_xO_4SeCl_2$ sample sets measured at 330 K. red line = 1st sample dataset, black line = repeat sample dataset..

The relationship between the Seebeck coefficient and carrier concentration in a material can be modelled using the equation:

$$S = \frac{8\pi^2 k_B^2 T}{3eh^2} m^* \left(\frac{\pi}{3(n_0 + n_d)}\right)^{\frac{2}{3}} \qquad \text{Eq. S2}$$

Where: S = Seebeck coefficient, m^* = carrier effective mass, n_0 = carrier concentration from the parent, n_d = carrier concentration from doping, and T = temperature. The number of charge carriers was calculated in $Bi_{4-x}Sn_xO_4SeCl_2$ ($0 \leq x \leq 0.08$) using the assumptions that: (1) m^* does not change with n_d, and (2) in the highest doping concentration, $n_d >>> n_0$ therefore $n_{total} \approx n_d$. The relationship between $n^{-2/3}$ and S is plotted in Figure S19. $Bi_{3.99}Sn_{0.01}O_4SeCl_2$ does not fit the expected linear trend illustrating the difference in carrier mass and number of charge carriers in this sample.

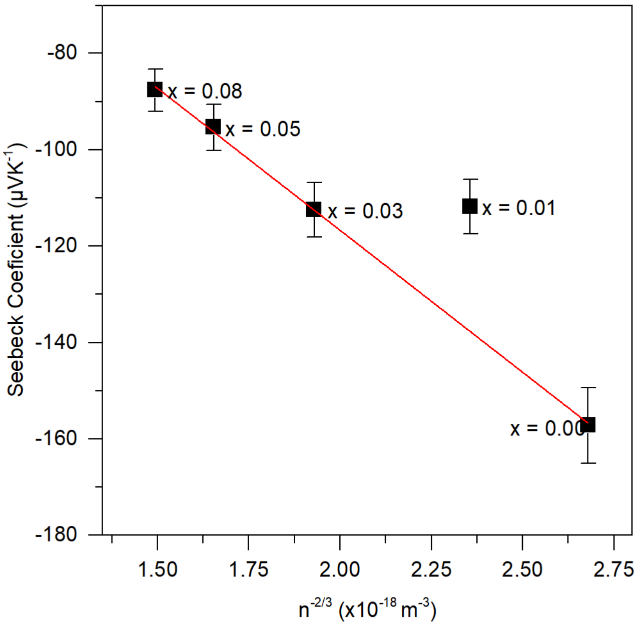

Figure S21. Plot of the Seebeck coefficient (S) against the calculated carrier concentration ($n^{-2/3}$) in $Bi_{4-x}Sn_xO_4SeCl_2$ using Equation S2.

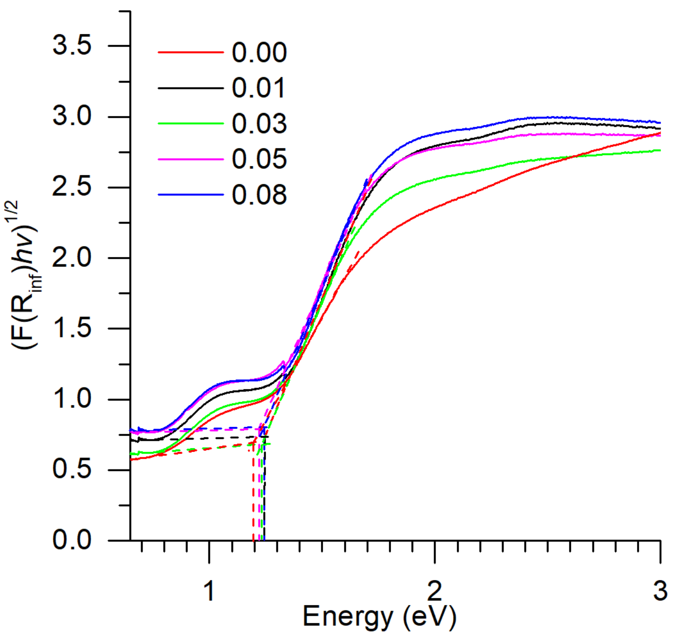

Figure S22. Tauc plot of $Bi_{4-x}Sn_xO_4SeCl_2$ samples measured by diffuse-reflectance UV-Vis Spectroscopy.

4. References

1. Brese, N. E.; O'Keeffe, M. Bond-Valence Parameters for Solids. *Acta Crystallogr. Sect. B.* **1991**, *47* (2), 192–197. https://doi.org/10.1107/S0108768190011041.
2. Gibson, Q. D.; Manning, T. D.; Zanella, M.; Zhao, T.; Murgatroyd, P. A. E.; Robertson, C. M.; Jones, L. A. H.; McBride, F.; Raval, R.; Cora, F.; Slater, B.; Claridge, J. B.; Dhanak, V. R.; Dyer, M. S.; Alaria, J.; Rosseinsky, M. J. Modular Design via Multiple Anion Chemistry of the High Mobility van Der Waals Semiconductor $Bi_4O_4SeCl_2$. *J. Am. Chem. Soc.* **2020**, *142* (2), 847–856. https://doi.org/10.1021/jacs.9b09411
